# Enhanced Chromatographic Determination of Nicotine in Human Plasma: Applied to Human Volunteers

**Published:** 2015-12

**Authors:** Bassam M. Ayoub, Samy Mohamady, Moataz S. Hendy, Mohamed M. Elmazar

**Affiliations:** 1 Department of Pharmaceutical Chemistry, Faculty of Pharmacy, British University in Egypt, El-Sherouk city, Cairo, Egypt;; 2 Department of Pharmacology, Faculty of Pharmacy, British University in Egypt, El-Sherouk city, Cairo, Egypt

**Keywords:** Nicotine, human plasma, UPLC, Liquid liquid extraction, vacuum evaporation, human volunteers

## Abstract

Development of enhanced UPLC-UV method for determination of nicotine in human plasma was achieved on a Symmetry^®^ C_18_ column (100 mm × 2.1 mm, 2.2 μm) applying isocratic elution based on Methanol: Acetonitrile: Phosphate Buffer (pH: 2.7) with the ratio (20:30:50, *v/v/v)* as a mobile phase. The ultraviolet detector was operated at 260 nm. The mobile phase was pumped through the column at a flow rate of 0.2 mL min^-1^. The column temperature was adjusted to 50ºC and the injection volume was 2 μL. Quinine was selected as an internal standard (IS) due to its structure similarity to nicotine having basic pyridine ring to optimize the liquid liquid extraction procedure using diethyl ether coupled with vacuum evaporation at 40°C. Validation parameters for nicotine were found to be acceptable over the concentration range of 2.5-50 ng ml^-1^. The application of the proposed method on four healthy human volunteers was approved by the ethical committee. The study was carried out under fasting conditions and the concerned subjects were informed about the objectives and possible risks involved in the study. The proposed method proved to be simple and fast which is a major advantage to analyze large number of samples per day using the accelerated vacuum evaporation technique. The method showed satisfactory data for all the parameters tested within the limits for bioanalytical assays. The lower limit of quantification (LLOQ) permits the application of the method for further pharmacological and clinical studies.

## INTRODUCTION

**Figure 1:**
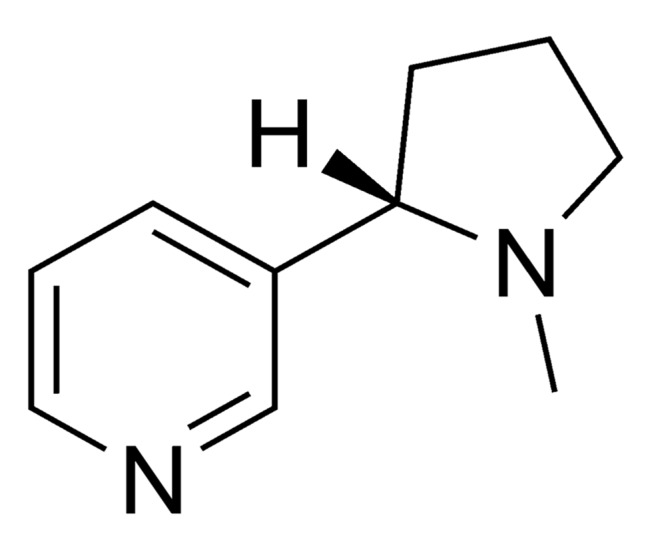
Chemical structure of nicotine.

Nicotine, (S) -3- [1-methylpyrrolidin-2-yl] pyridine (Figure 1) is a potent alkaloid that constitutes about 3% of the dry weight of tobacco found in cigarettes. Nicotine’s addictive nature includes psychoactive effects, relapse after abstinence and physical dependence (1). To the best of the authors’ knowledge after literature review, there are no published UPLC-UV methods for sensitive determination of nicotine in human plasma. Many HPLC-UV methods (1-8) have been reported for nicotine determination lacking the UPLC major advantages of consuming less solvent and less time.

The aim of the work is to develop the first enhanced UPLC-UV method for determination of nicotine in human plasma using Quinine as internal standard (IS) and its application on human volunteers. The method is suitable for further pharmacological studies with the advantages of enhanced chromatographic behavior, enhanced extraction procedure and short analysis time comparative to the other routine HPLC-UV methods reported in the literature (2-8).

## EXPERIMENTAL

### Instrumentation

The liquid chromatography consisted of a Thermo Fisher UPLC Model Ultimate 3000 (USA), a Symmetry^®^ C_18_ column (100 mm × 2.1 mm, 2.2 μm) equipped with a Diode Array detector (DAD-3000RS, USA) and an auto sampler (WPS-3000TRS, USA). An Elmasonic S60H (Germany) was used for the degassing of the mobile phase. Jenway (UK) digital pH meter was used. Vacuum evaporator Christ^®^ (S/N 37399708, Germany), Vacuum pump vacwbrand^®^ (DVP2C-TYR012, Germany), Vortex Velp Scientifica^®^ (F202A0280, S/N 265349, Europe) and Centrifuge Hettich^®^ (T1.6AH/250V, S/N 012444807, Germany) were used.

### Human plasma, Reagents and reference samples

Human plasma was supplied from Vacsera (Egypt). Nicotine certified to contain 99.80 % was purchased from EMD Millipore Corporation (Germany). HPLC grade acetonitrile and methanol were purchased from Fisher Scientific (UK). Diethylether was purchased from Sigma Aldrich (Germany). Bi-distilled water was produced in-house (POLNA, DE 10, Poland). PTFE Membrane Filter, 47 mm, 0.20 μm (UK) were used. Orthophosphric acid (85%) was purchased from VWR Chemicals (UK). HPLC grade potassium dihydrogen phosphate was purchased from Sigma Aldrich (Germany).

### Preparation of working solutions

Different nicotine working solutions were prepared in distilled water (0.25, 0.5, 1, 1.5, 2, 3, 4, 4.5 and 5 μg/mL) equivalent to (2.5, 5, 10, 15, 20, 30, 40, 45 and 50 ng/10 μL). While quinine IS working solution was prepared in methanol (20 μg/mL) equivalent to (100 ng/5 μL).

### Procedure

**Calibrators and chromatographic conditions.** Series of 1 ml plasma samples was spiked with 10 μL of different nicotine working solutions (2.5, 5, 10, 20, 30, 40 and 50 ng/10 μL) separately followed by spiking with 5 μl of Quinine working solution (100 ng/5 μL) then alkalinized with 0.1 ml of 10 M NaOH before adding the organic extracting phase (2 ml Diethyl ether) and vortex for 3 minutes at 3000 RPM, The samples centrifuged for 12 minutes at 6000 RPM then the upper layer was separated (diethyl ether). 5 μl concentrated HCL was added to the separated diethyl ether layer then vortex was used for 3 min at 3000 RPM followed by vacuum evaporated at 40°C - 1400 RPM till complete dryness of the sample, Reconstituted with 0.5 ml mobile phase, vortex for 3 minutes at 3000 RPM followed by filtration using syringe filter and finally transferred to the vials in the auto sampler and 2 micro liters were injected into the C_18_ column.

Chromatographic determination was achieved on a Symmetry^®^ C_18_ column (100 mm × 2.1 mm, 2.2 μm) applying an isocratic elution based on Methanol: Acetonitrile: Phosphate Buffer (pH: 2.7) with the ratio (20:30:50, *v/v/v)* as a mobile phase. The ultraviolet detector was operated at 260 nm. The buffer solution was filtered through 0.2 μm membrane filter and degassed for 30 min in an ultrasonic bath prior to its use. The mobile phase was pumped through the column at a flow rate of 0.2 mL min^-1^. The column temperature was adjusted to 50ºC and the injection volume was 2 μL. The calibration curve for nicotine was obtained, representing (the relation between the peak area ratio of nicotine to the internal standard) and (the corresponding nicotine concentration), the regression equation was calculated.

**Quality control samples (QC) for the bioanalytical validation.** Series of 1 ml plasma samples was spiked with 10 μL of the different nicotine working solutions (5, 15 and 45 ng/10 μL) separately followed by spiking with 5 μl of Quinine working solution (100 ng/5 μL) and the procedure mentioned under [Calibrators and chromatographic conditions] was repeated. Repeatability was assessed by analysis of replicates of three different low, medium and high QC levels (LQC, MQC and HQC) on the same day and on three separate days. The accuracy of the method expressed in terms of bias (percentage deviation from true value) was assessed by calculation of recovery percent of the QC samples.

Extraction recovery was calculated by comparing the AUP of extracted QC samples to the AUP of the same unextracted QC samples. Carry over was studied by injection of blank plasma after high concentration QC sample. Matrix effect was studied by finding the ratio of AUP of QC samples in the presence of the matrix to AUP of QC samples in absence of the matrix. Dilution integrity was assessed by comparing the serially diluted QC samples with directly spiked samples. Finally, stability studies were assessed by storing the QC samples for 24 h at room temperature. Post preparative stability was assessed by keeping the QC samples in the auto sampler for 24 h while freeze and thaw stability was carried out by freeze at -20 and unassisted thaw of the QC samples.

**Application to biological samples (human volunteers).** Samples from four healthy human volunteers were analyzed by the same previous proposed extraction method under [Calibrators and chromatographic conditions] five minutes after smoking of one cigarette. The blood samples (4 mL of each sample) were collected in EDTA tubes and centrifuged immediately at 3000 rpm for 5 minutes, one mL of the plasma was separated and then the previous procedure under [Calibrators and chromatographic conditions] was applied. The study was carried out under fasting conditions and the concerned subjects were informed about the objectives and possible risks involved in the study. The study design was approved by the ethical committee.

## RESULTS AND DISCUSSION

### Optimization of sample preparation and major advantages over the reported methods

**Figure 2:**
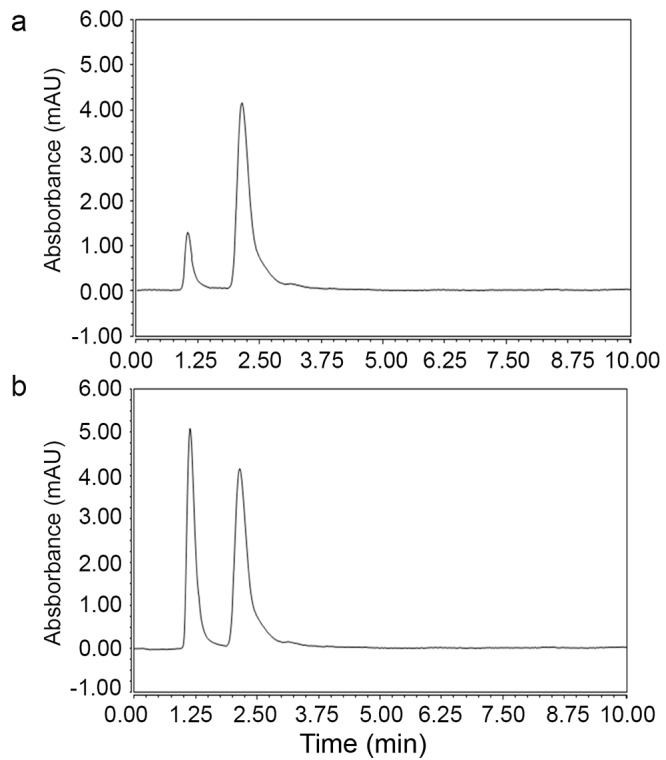
a) UPLC chromatogram of nicotine at its lower limit of quantification LLOQ (2.5 ng mL^-1^) at 1.1 min in the presence of Quinine internal standard IS (100 ng mL^-1^) at 2.2 min; b) UPLC chromatogram of nicotine in human volunteer sample (7.6 ng mL^-1^) at 1.1 min in the presence of Quinine internal standard IS (100 ng mL^-1^) at 2.2 min.

Quinine was selected as an internal standard (IS) due to its structure similarity to nicotine having basic pyridine ring to optimize the liquid liquid extraction procedure as the lone pair on the pyridine nitrogen is not involved in resonance and not included within the aromatic system of the ring so it will be available increasing the basicity of both nicotine and quinine. Liquid liquid extraction using diethyl ether was better than dichloromethane and the other solvents reported in the literature. Vacuum evaporation was the best technique selected for evaporation till dryness. Reconstitution with the mobile phase showed the best results in comparison to distilled water and methanol. Different time intervals were investigated for the vortex and centrifugation till optimization of the method. Different normalities of NaOH and HCl were tried to ensure the optimum migration of nicotine and IS to the diethylether layer. The first enhanced UPLC-UV method for determination of nicotine in human plasma using Quinine as internal standard (IS) is characterized with high throughput analysis (Figure 2) over the routine HPLC-UV methods reported in the literature (2-8) and applicable to nicotine analysis from human volunteers (Figure 2).

### Method development for the chromatographic method

Several parameters were considered to enhance peak intensity. C_18_ column was suitable for the estimation. Applying flow rate more than 0.2 increased back pressure which is not suitable for determination of biological samples. Isocratic elution based on Methanol: Acetonitrile: Phosphate Buffer (pH: 2.7) with the ratio (20:30:50, *v/v/v)* as a mobile phase was suitable for the investigation with no need to the gradient mode. The ultraviolet detector was operated at 260 nm which was sensitive for both nicotine and quinine (Figure 2). The buffer solution was filtered through 0.2 μm membrane filter and degassed for 30 min in an ultrasonic bath prior to its use. The column temperature was adjusted to 50°C and the injection volume was 2 μL.

### Validation parameters

**Linearity.** The calibration curve was constructed by plotting (peak area ratio of nicotine to quinine IS) versus (nicotine concentrations) in plasma (2.5-50 ng/mL). The calibration curve was linear in the studied range and the regression parameters were computed and found to be Y= 0.0509 X – 0.011 where Y is the ratio of nicotine peak area to the internal standard peak area and X is nicotine concentration in plasma. The correlation coefficient was found to be 0.9994. The lower limit of quantification (LLOQ) was 2.5 ng/mL and the standard error of the estimation (STEYX) was equal to 0.04256.

**Accuracy and precision.** Accuracy demonstrates the closeness of the results obtained to the true value while precision illustrate the closeness of individual measures when the procedure is applied repeatedly (9). It was performed by repeated analysis of three concentrations representing the entire range of the calibration. Close to the lower limit of quantification LLOQ (low QC), Close to the middle of the calibrators (medium QC) and close to the upper calibration curve range (high QC). The accuracy of the method expressed in terms of bias (percentage deviation from true value). The mean values are within 15% of the actual values for the QC samples (bias ranging from 7.85 % to 14.45 %). The accuracy % values (72.38- 91.69 %) and the intra and interday standard deviation values (6.84 – 9.96) were within the acceptable range.

**Extraction recovery.** The peak areas of extracted LQC, MQC and HQC were compared to the absolute peak area of the unextracted samples. Recoveries were found to be higher than 70%. The average recovery was found to be 84.15%.

**Carry-over.** Carry over was assessed by injecting blank samples after a high concentration sample. Average recoveries were less than 20% of the lower limit of quantification (ranging from 13.85 % to 17.37 %).

**Matrix effect.** Matrix effect is the ratio of the peak areas in the presence of matrix to the ratio in absence of matrix (9). The relative standard deviation of peak area ratios (analyte/IS) was ranging from 1.75 % to 2.43 % indicating no significant matrix effects.

**Dilution integrity.** Dilution integrity should be demonstrated by diluting sample with blank matrix to ensure that sample could be diluted with blank matrix (two and four folds) without affecting the final concentration. The replicates showed precision below 15%.

Stability. Short term stability was determined by keeping aliquots of LQC, MQC and HQC samples for period of 24h at room temperature and then analyzed. Post-Preparative Stability was examined by keeping aliquots of QC samples in the auto sampler at 25°C for one day. Freeze and Thaw Stability was determined after storing samples at -20°C for 24 hours and thawed at room temperature. Stability was accepted as the concentration change was less than 15% of the actual values.

### Application of the developed method on human volunteers

The application of the proposed method was applied successfully on four healthy human volunteers (Figure 2) after approval by the ethical committee. The concerned subjects were informed about the objectives and possible risks involved in the study. Moving from HPLC to UPLC has major advantages of consuming less solvent and less time (10) which is critical for biological samples analysis to enable analysis of large number of samples per day.

## CONCLUSION

The first proposed UPLC-UV method proved to be accurate for determination of nicotine in human plasma. The method showed satisfactory data for all the parameters tested within the limits for bioanalytical assays. The lower limit of quantification permits the application of the method on human volunteers and suitable for further pharmacological studies.
